# 

**Published:** 1897-11

**Authors:** 


### PENNSYLVANIA STATE BOARD OF VETERINARY MEDICAL EXAMINERS.

The regular December meeting will be held in Philadelphia on December 14, 1897, to examine all applicants for license and to answer all other matters placed before the Board.

W. Horace Hoskins, President. S. J. J. Harger, Secretary.

### PERSONAL.

He Turned Up, but was Attired in Military Garb.—There are some individuals whose presence or absence at any representative gathering of the profession or business to which they belong gives occasion for remark. Such was the case with one of our prominent Eastern members, whose conspicuous figure was so much missed from the thirty-fourth annual convention of the United States Veterinary Medical Association, held in Nashville, Tenn., the correct date of which he was, we have no doubt, officially notified; but on account of some valid reason, best known to himself, did not put in an appearance "on time." Although some thirty or forty days late, however, he did turn up in the Centennial City, but attired in the chevrons of a sergeant-major of Troop C, N. G., N. Y. Just how long our gallant military confrère had frequented the beautiful and picturesque grounds of our Southern White City we are not prepared to say, but he was found on the streets of Cairo, where he had, no doubt, been testing his equestrian powers on some of the "ships of the Eastern desert." There was evidence, also, that our worthy friend had bestowed considerable patronage upon various other sights and frivolities to be seen and enjoyed on "Vanity Fair." Compassion for our friend touched a respondent chord in our hearts, and we saw him safely off from the union depot, with the train headed East. In this act of fraternal consideration the profession are under a debt of gratitude to the veterinarians of Nashville. So left us Dr. W. H. Pendry, of Brooklyn, N. Y., with the parting ejaculation on his lips: "Just tell them that you saw me."

Dr. W. L. Williams, of Ithaca, N. Y., addressed the Tompkins County Medical Association, in October, on "Irrigation of the Bronchi."

We are glad to know of the return of Dr. Nelson P. Hinkley, of Buffalo, to duty again after a successful operation.

Dr. George Jobson, Jr., of Franklin, Pa., has entered the medical department of the Columbia University at Washington, D. C., for the completion of a course in human medicine. Dr. Jobson will fill the chair of anatomy at the veterinary department of this institution during the sessions of 1897 and 1898.

Veterinarian H. R. Cleveland, of Danville, Quebec, is much interested in the breeding of standard horses in connection with "Rock Farm."

Dr. Hobart A. Hare, editor of the *Therapeutic Gazette*, has sufficiently recovered from his recent attack of typhoid to hazard a trip to Bristol, R. I., to recuperate.

Dr. Walter L. Hart, City Veterinarian of Philadelphia, had the misfortune in October of losing one of his children.

One afternoon along the drive of Central Park, N. Y., we noticed Dr. T. G. Sherwood seated behind a pair of heavy-harness horses attached to a brake-wagon; Dr. Buckley with a speedy one waiting for a brush at every favorable point; Dr. W. D. Critcherson taking a quiet afternoon drive; Dr. H. D. Gill with his fast pacer and trotter, harnessed to a bicycle road-wagon, and looking for fresh laurels on the road with his fast-gaited pair.

Governor Hastings, of Pennsylvania, has reappointed Drs. J. C. McNeil, of Pittsburg, and Harry Walters, of Wilkesbarre, members of the Pennsylvania State Board of Veterinary Medical Examiners.

Dr. W. L. Thayer, formerly of Marlborough Depot, N. H., has changed his place of residence to Worcester, Mass.

Dr. L. A. Merillat, of the McKillip Veterinary College, was called upon for expert testimony by the defense in the celebrated Luetgert murder case. Dr. Merillat's testimony was to the effect that the femur-bone found was that of a hog instead of a woman, as the prosecution tried to prove.

Dr. Frederick L. Felber, of Baltimore, Md., was a visitor to Philadelphia in October.

Dr. N. S. Mayo, late of Manhattan, Kansas, who had matriculated at the New York State Veterinary College, has been called to Storr's Agricultural College, Conn.

Secretary Edge, of the State Board of Agriculture of Pennsylvania, with State Veterinarian Pearson, of the Live-stock Sanitary Board, were visitors to the Department of Agriculture at Washington, D. C., in October, looking to the enforcement of the new regulations in Pennsylvania governing the introduction of dairy cattle as to tuberculosis.

Dr. W. S. Underhill, of Media, Pa., recently saved the life of a little two-year-old tot from drowning while on his professional rounds.

Dr. W. Horace Hoskins, one of the editors of the JOURNAL, will lecture to the veterinary students of the University of Pennsylvania during the present college year on veterinary jurisprudence and ethics.

Veterinarian T. B. Pote, Sanitary Inspector of the Board of Health of Terre Haute, Indiana, read a paper in October before the Vigo Medical Society on "Some Dangers of Milk."

Dr. F. deM. Bertram, of Newport, Rhode Island, was among the list of applicants before the New York State Board of Veterinary Medical Examiners.

Dr. R. T. Stanclift, of Americus, Ga., a graduate of Ontario Veterinary College, is among the students of the New York State Veterinary College at Ithaca, N. Y.

Dr. W. T. Monsarrat, graduate of the Ontario Veterinary College, class of '89, is now employed by the Board of Health of Honolulu, Hawaiian Islands, as meat-inspector and inspector of dairy-cattle.

Veterinarian W. T. Frady, of Marietta, Ind., met with a severe loss by fire in October, which destroyed the contents of his stable, including forty-three valuable horses in training. The total loss will reach $25,000.

Prof. R. S. Huidekoper stills spends Friday to Monday at Newport, Rhode Island, looking after his professional work at that watering resort.

The veterinary hospital of Dr. B. M. Freed, of Sharon, Pa., was badly damaged by fire in October.

Edward T. Reichert, M.D., Professor of Physiology in the Medical Department, succeeds Dr. W. S. Carter in the same work in the Veterinary Department of the University of Pennsylvania.

Dr. Alexander Glass, of Philadelphia, has matriculated at the Jefferson Medical College for a course in human medicine.

Dr. William Dougherty, of Baltimore, Md., was a visitor to the JOURNAL office in October, spending a part of his day in the Quaker City at the Veterinary Department of the University of Pennsylvania.

Dr. Theobald Smith delivered the introductory address at the opening of the Veterinary Department of Harvard University in October.

Dr. Cooper Curtice, of Moravia, N. Y., has recently been associated with the Massachusetts Cattle Commission in the investigation of Texas-fever within the borders of the Bay State.

Dr. Paul Fischer, of Ogden, Utah, has succeeded Dr. N. S. Mayo at the State Agricultural College at Manhattan, Kansas.

The Board of Trustees of Clemson College, S. C., have granted an eight months' leave of absence to Dr. W. E. A. Wyman, which will be partly devoted to clinical medicine and bacteriological and pathological researches. His clinical work will be done at McKillip College.

Dr. William H. Pendry, of the Thirteenth Regiment of Brooklyn, was a visitor to the Nashville Exposition in October on Brooklyn day. On his return home he visited a number of veterinarians in Washington, Baltimore, and Philadelphia. His visit to Philadelphia included a trip to the University and the JOURNAL office.

Dr. A. J. Thompson, of Terre Haute, Indiana, is in the hands of the court on the charge of perjury relative to an alleged payment of an insurance premium to a company he represents in the above city. The evidence of holding is that such payment was never paid as alleged.

Dr. A. H. Wallace, of Turkey, N. J., was a recent applicant for license before the New York State Board of Veterinary Medical Examiners.

Prof. Wesley Mills, of Montreal, started for a trip abroad in October, Leipsic, Germany, to be his ultimate destination.

Dr. Thomas Castor, of Frankford, Philadelphia, has received an appointment as meat-inspector under the Bureau of Animal Industry, and directed to report at Buffalo, N. Y., for duty.

Dr. W. C. Rayen, of Nashville, has been granted a year's leave of absence from duty by the Bureau of Animal Industry at Washington, D. C.

Dr. L. A. Greiner, of Indianapolis, is a frequent contributor of answers to the inquiry-column of the *Jersey Bulletin* as to diseases of cattle.

Drs. W. G. Clark, of Beaver Dam, and H. P. Clute, State Veterinarian of Wisconsin, have formed a copartnership under the firm name of Clute & Clark, and located at Marinette in the Badger State.

The veterinary inspectors at the Chicago horse-show, November 1st to 6th, were Drs. M. H. McKillip, Frank T. McMahon, and B. A. Pierce.

Dr. B. T. Smelzer, Secretary of the New York State Board of Health, has issued a catechism on tuberculosis for distribution throughout the State to all inquiries as to the nature and character of this disease.

Prof. A. Smith, of Toronto, Canada, is one of the stewards of the Ontario Jockey Club, and fills the post well.

Dr. C. C. McLean, of Meadville, Pa., exhibited three very good horses in several classes and won some prizes at the horse-show in Pittsburg during the month of October.

Dr. C. S. McKenna, of Washington, has been spending his vacation at a sanitarium in Michigan.

Dr. C. Barnwell Robinson, President of the District of Columbia Veterinary Medical Association, made part of his mission to Nashville the securing of the meeting of the U S. V. M. A. for 1898 at Washington. The press of Washington and the Commissioners of the District favor the Capitol City for the convention Mecca for 1898.

Dr. G. M. Brumbaugh, Assistant Chief of the Bureau of Animal Industry, is making a personal investigation for the Washington authorities of the disease among cattle near DuBois, Pa., which has been reported as anthrax.

Dr. W. L. Zuill, of Philadelphia, has recently made a very large contribution of important and valuable pathological specimens to the New York State Veterinary College.

Prof. R. S. Huidekoper was recently the chief participant in a bicycle collision on Fifth Avenue, New York City, whereby he found himself unceremoniously at the feet of a lady in a drag. After extricating himself from this embarrassing position, making due apology for his uninvited call, gathering up his broken wheel, and calling a cab, he made his way home in a four-wheeler, but little the worse bodily for his experience.

Dr. T. E. White, State Veterinarian of Missouri, started in early September for a two months' circuit of the Farmers' Institutes.

Dr. Leonard Pearson, State Veterinarian of Pennsylvania, addressed several of the Farmers' Institutes of the Keystone State during the month of September.

Dr. A. K. Robertson, of Brooklyn, was one of the judges of horses at the New York State Fair.

Dr. M. H. Reynolds was one of the judges at the Minnesota State Fair this year, and will judge the horses at a number of county fairs during the fall. The doctor has always been a close student of horse form and proportion, and seems to be winning reputation in the Northwest as an expert judge of good horses in the show-ring.

Veterinarian T. Earle Budd, of Woodbury, N. J., was defeated for the nomination of Coroner of Gloucester County in September.

